# The role of chest CT quantitative pulmonary inflammatory index in the evaluation of the course and treatment outcome of COVID-19 pneumonia

**DOI:** 10.1038/s41598-021-87430-5

**Published:** 2021-04-08

**Authors:** Song Peng, Jinqing Chen, Wendy Zhang, Bangjun Zhang, Zhifeng Liu, Lang Liu, Zhaofeng Wu, Rui Fu, Xiuhua Li, Fajin Lv

**Affiliations:** 1 Department of Radiology, Chongqing Health Center for Women and Children, 120 Longshan Road, Chongqing, 400021 China; 2 Department of Radiology, Xiaochang First People’s Hospital, 1 Station Front Road, Xiaochang, 432900 Hubei China; 3 New York Medical College, Valhalla, NY 10595 USA; 4 Department of Radiology, First Affiliated Hospital of Chongqing Medical University, 1 Youyi Road, Chongqing, 400016 China

**Keywords:** Diseases, Medical research, Signs and symptoms

## Abstract

To explore the clinical application value of chest CT quantitative pulmonary inflammation index (PII) in the evaluation of the course and treatment outcome of COVID-19 pneumonia. One hundred and eighteen patients with COVID-19 pneumonia diagnosed by RT-PCR were analyzed retrospectively. The correlation between chest CT PII, clinical symptoms and laboratory examinations during the entire hospitalization period was compared. The average age of the patients was 46.0 ± 15 (range: 1–74) years. Of the 118 patients, 62 are male (52.5%) and 56 are female (47.5%). Among them, 116 patients recovered and were discharged, 2 patients died, and the median length of hospital stay was 22 (range: 9–41) days. On admission, 76.3% of the patients presented with fever, and the laboratory studies showed a decrease in lymphocyte (LYM) count and an increase in lactate dehydrogenase (LDH) levels, C-reactive protein (CRP) levels, and erythrocyte sedimentation rate (ESR). Within the studies’ chest CTs, the median number of involved lung lobes was 4 (range: 0–5) and the median number of involved lung segments was 9 (range 0–20). The left lower lobe and the right lower lobe were the most likely areas to be involved (89.0% and 83.9%), and 84.7% of the patients had inflammatory changes in both lungs. The main manifestations on chest CT were ground glass opacities (31.4%), ground glass opacities and consolidation (20.3%), ground glass opacities and reticular patterns (32.2%), mixed type (13.6%), and white lungs (1.7%); common accompanying signs included linear opacities (55.9%), air bronchograms (46.6%), thick small vessel shadows (36.4%), and pleural hypertrophy (13.6%). The chest CT at discharge showed complete absorption of lesions in 19 cases (16.1%), but not in the remaining 99 cases. Lesions remained in a median of 3 lung lobes (range: 0–5). Residual lesions remained in a median of 5 lung segments (range: 0–20). The residual lesions mainly presented as ground glass opacities (61.0%), and the main accompanying sign was linear opacities (59.3%). Based on chest CT, the median maximum PII of lungs was 30.0% (range: 0–97.5%), and the median PII after discharge in the patients excluding the two deaths was 12.5% (range: 0–53.0%). PII was significantly negatively correlated with the LYM count and significantly positively correlated with body temperature, LDH, CRP, and ESR. There was no significant correlation between the PII and the white blood cell count, but the grade of PII correlated well with the clinical classification. PII can be used to monitor the severity and the treatment outcome of COVID-19 pneumonia, provide help for clinical classification, assist in treatment plan adjustments and aid assessments for discharge.

## Introduction

Since December 2019, many cases of viral pneumonia have been detected in Wuhan, Hubei Province of China. The epidemic has rapidly spread in mainland China and other countries and regions. On February 10, 2020, the International Committee of Taxonomy officially named the virus that caused the disease SARS-CoV-2 (Severe Acute Respiratory Syndrome Coronavirus 2)^1^. On February 11, 2020, the WHO officially named the disease caused by the novel coronavirus as COVID-19 (Corona Virus Disease-19)^2^. COVID-19 pneumonia is clinically characterized by fever, dry cough, and fatigue. Most patients experience a decrease in lymphocyte (LYM) count and an increase in C-reactive protein (CRP) levels and erythrocyte sedimentation rate (ESR). The diagnosis of COVID-19 pneumonia is based on the patient’s epidemiological history and the corresponding symptoms, a positive result on SARS- CoV-2 nucleic acid tests by real-time fluorescent reverse transcription-polymerase chain reaction (RT-PCR), and the typical manifestations of COVID-19 pneumonia on chest CT, including interstitial inflammation and small patchy shadows that may progress to lung infiltrates and ground glass opacities^3^.

Chest CT plays an important role in screening suspected patients, evaluating for signs of COVID-19 pneumonia, and assessing requirements necessary for discharge^4^. The condition of patients with COVID-19 pneumonia changes rapidly, and it is often necessary to perform multiple CT examinations to evaluate the lung lesions throughout the treatment process^5,6^. Chest CT can be objectively used to reflect the changes of lesions in different stages of COVID-19 pneumonia, which is more conducive to clinical protocol and adjustments to the treatment plan. At present, there are many studies have focused on the pattern and semi-quantitative CT findings to diagnose and evaluate the severity of COVID-19 pneumonia. However, a few quantitative analyses of the CT features have been reported. The semi-quantitative methods used for COVID-19 pneumonia are as follows: a calculation method using software that automatically sketches volume^7^ and a calculation method for evaluating the degree of involvement according to 5 lobes or 6 zones of lungs^8,9^. Therefore, we intended to use the pulmonary inflammation index (PII), which is a score system based on the distribution of pulmonary inflammatory lesions on chest CT, to quantitatively evaluate the pulmonary inflammation of COVID-19 pneumonia. Then, combined with clinical symptoms and laboratory index changes, we further analyzed the relationship between the PII and the clinical course to evaluate the clinical application value of chest CT quantitative PII in assessing the course and treatment outcome of COVID-19 pneumonia.

## Materials and methods

All of the methods were carried out in accordance with relevant guidelines and regulations. This study was approved by the ethics committee at Xiaochang First People's Hospital and Chongqing Health Center for Women and Children. Informed consent was obtained from all the patients or their legal guardians if the patients are under 18 years.

### Subjects

The one hundred and eighteen patients who met the epidemiological history and clinical manifestations of COVID-19 pneumonia as confirmed by RT-PCR and who were admitted to the First People's Hospital of Xiaochang County, Hubei Province from January 22 to March 16, 2020 were retrospectively reviewed. The body temperature and related laboratory test results during hospitalization were recorded. The laboratory values studied include the white blood cell (WBC) count, the LYM count, lactate dehydrogenase (LDH) levels, CRP levels, and ESR. Every patient’s clinical classification corresponding to each CT examination was also recorded.

Clinical classification criteria^3^ were as follows: (1) Mild type: the clinical symptoms were mild, and no pneumonia manifestations in imaging. (2) Moderate type: patient displays fever, respiratory tract and other symptoms. Pneumonia can be seen on imaging. (3) Severe type: for adults, any of the following criteria: ① shortness of breath, RR ≥ 30 times/min; ② in resting state, oxygen saturation ≤ 93%; ③ arterial partial blood oxygen pressure (PaO_2_) / Fraction of inspiration oxygen (FiO_2_) ≤ 300 mmHg (l mmHg = 0.133 kPa). At altitudes above 1000 m, PaO_2_ / FiO_2_ should be corrected according to the following formula: PaO_2_ / FiO_2_ × [atmospheric pressure (mmHg) / 760]. For children, any of the following criteria: ① shortness of breath (< 2 months old, RR ≥ 60 times / min; 2–12 months old, RR ≥ 50 times / min; 1–5 years old, RR ≥ 40 times / min; > 5 Years old, RR ≥ 30 times / min); ② in resting state, oxygen saturation ≤ 92%; ③ moaning, deep recession of the suprasternal fossa, the suprasternal fossa, and intercostal spaces in inspiration, cyanosis, intermittent apnea; ④ drowsiness, convulsions; ⑤ refusal to eat or feeding difficulties, with signs of dehydration. (4) Critical type: those who meet one of the following criteria: ①respiratory failure requiring mechanical ventilation; ②shock; ③compounding organ failure that requires monitoring and treatment in ICU.

### Etiology examination

RT-PCR was used to detect SARS-CoV-2 nucleic acid in oropharyngeal and nasopharyngeal swab specimens.

### Chest CT examination

Chest CT examination was performed using GE64 row 128-slice CT scanning equipment (GE HANGWEI Medical System, Beijing, China), from the level of the thoracic entrance to the level of the diaphragm, and completed at the end of inspiration. The scanning parameters were as follows: tube voltage 120 kV, tube current 250 ~ 450 mA, layer thickness 10 mm, and layer spacing 5 mm. At the end of scanning, a thin layer image with a layer thickness of 1.25 mm and a layer distance of 1.25 mm is automatically reconstructed and recorded as DICOM image data. The reconstruction algorithm used is the lung algorithm with a field of view of 500 mm × 500 mm and a matrix of 512 × 512. Image browsing and multi-plane reconstruction were performed using GE AW VolumeShare software (Version number:4.6;http://www.gehealthcare.com/products/advanced-visualization/platforms/aw-server); images of the lungs (window width 1600 HU, window level -500) and the mediastinum (window width 350 HU, window level 50) were also observed using the same software.

### Image analysis

Two radiologists with more than 10 years of experience in chest imaging diagnosis jointly analyzed the image characteristics and used a double-blind method for quantitative CT scoring. For cases with controversial double-blind scoring results, three radiologists with more than 10 years experience in chest imaging diagnosis jointly reviewed and evaluated the final score.

For each patient, the characteristics of the chest CT scan were evaluated. The main features included the following: (1) ground glass opacities; (2) ground glass opacities and consolidation; (3) ground glass opacities and reticular patterns; (4) mixed type (ground glass opacities with consolidation and reticular patterns); (5) white lungs. Other signs that were noted include the following: (1) linear opacities; (2) air bronchograms; (3) nodular shadow; (4) thickened small blood vessel shadow; (5) pleural hypertrophy; (6) enlarged mediastinal lymph nodes; (7) pleural effusion.

Quantification of PII is simple and easy to evaluate: it refers to scoring based on the distribution and extent of the lesions on chest CT. The score for lesion distribution is based on the number of lung segments in the lesion distribution, one point for each segment. For convenience of clinical measurement, symmetrical analysis of left and right lungs was performed. The posterior apical segment of the left upper lobe is divided into two score segments, and the inner anterior basal segment of the left lung is also divided into two score segments. The divisions create a total of 20 score segments from the left and right lobes, and each division is scored separately. Scoring depends on the volume ratio of the segment occupied by the lesion: a ratio exceeding 50% equals 1 point; a ratio less than 50%, 0 points. A maximum of 20 points is possible. PII = (lesion distribution score + volume score)/total score (40) × 100%. This quantitative assessment can be used to quantify the load of inflammatory lesions. The score combined with clinical data can be used to evaluate the extent of lesion changes for patients with COVID-19 pneumonia. The grades are as follows: grade 0: no obvious lesions in the lungs (PII = 0%), grade I (PII = 1–25%), Grade II (PII = 26–50%), Grade III (PII = 51–75%), and Grade IV (PII = 76–100%).

### Time window for chest CT reevaluation

For newly diagnosed patients with typical clinical manifestations (had epidemiological history, were clinically characterized by fever, dry cough, and fatigue) and a positive detection of SARS-CoV-2 nucleic acid but a negative initial chest CT, it is recommended to reevaluate the chest CT in 3–5 days to observe if any new disease findings have emerged in the lungs. For those with atypical clinical manifestations, but with typical radiological features of COVID-19 pneumonia, it is recommended to perform a repeat chest CT in 5–7 days to observe the changes in the lesions. It is also recommended to repeat chest CT in 5–7 days for patients without critical illness^4^.

### Treatment and discharge criteria

All patients were hospitalized and received a standard treatment. For mild type and moderate type patients, Strengthening support therapy and antiviral therapy; For severe type and critical type patients, On the basis of symptomatic treatment, complications should be proactively prevented, underlying diseases should be treated, secondary infections also be prevented, and organ function support should be provided timely.

Discharge criteria are defined as follows: (1) body temperature returned to normal for more than 3 days; (2) respiratory symptoms improved significantly; (3) chest CT showed a significant improvement in acute exudative lesions; (4) two consecutive oropharyngeal and nasopharyngeal swab specimens tested negative for nucleic acid (sampling time at least 24 h apart)^3^.

### Statistical methods

SPSS 25.0 statistical software was used for statistical analysis. Normal distribution data was indicated as mean ± standard deviation. Non-normal distribution data was reported as median (minimum value, maximum value). A chi-square test was applied for the analysis of the quantitative and enumerated data; a Mann–Whitney U test was used for PII comparison. Spearman correlation analysis was used to compare the correlation of chest CT PII with clinical symptoms and laboratory test results. A *P* value of less than 0.05 was defined as statistically significant. Bland–Altman tests were used to assess the reliability and reproducibility of CT findings between the two radiologists.

## Results

### Baseline characteristics

The average age of the patients was 46.0 ± 15 years (range: 1–74 years). Among the 118 patients, 62 were males (52.5%) and 56 were females (47.5%). After a median of 22 (range: 9–41) days of hospitalization, 116 patients recovered and were discharged from the hospital, and 2 patients died. The two patients died at the ages of 68 and 39 years, respectively. The patient who died at age 68 did not have other diseases, but the patient who died at age 39 had noted comorbidities of diabetes and uremia. During the hospital stay, a total of 583 chest CT examinations were performed on these 118 patients. The median number of CT examinations performed per patient was 5 (range: 3–6), and the median CT examination interval was 4.5 (range: 3–8) days. Based on the classification criteria, 1 case was classified as mild (0.8%), 111 cases were classified as moderate (94.1%), 4 cases were classified as severe (3.4%), and 2 cases were classified as critical (1.7%). The two patients that died were both classified as critical types (Table 1).

**Table 1:** Baseline characteristics of the patients with COVID-19.

	All patients (n = 118)
Age (years old)	46 ± 15 (1–74)
**Sex**	
Male	62 (52.5%)*
Female	56 (47.5%)*
Hospitalization time (days)	22 (9–41)
Recovery	116 (98.3%)
Death	2 (1.7%)
Number of CT examinations	5 (3–6)
Average time between CT examinations (days)	4.5 (3–8)
Mild type	1 (0.8%)
Moderate type	111 (94.1%)
Severe type	4 (3.4%)
Critical type	2 (1.7%)

*No significant difference was observed between the sex of two groups, *p* = 0.435.

### Temperature and laboratory test results

When the patients were admitted to the hospital, 76.3% of them had the temperature higher than 37.2℃, with the median body temperature at 37.8℃(range: 36.0–39.6℃). The temperature had returned to normal (below 37.2℃) in all patients by the time they were discharged from the hospital, with a median body temperature of 36.5℃(range: 36.0–37.2℃) at time of discharge. The median number of WBC upon admission was 4.52 × 10^9^/L (range: 0.83–14.69 × 10^9^ /L). 5.7% of the patients had a WBC count above normal range and 27.1% of the patients had a WBC count below normal range. The median number of WBC upon discharge was 5.69 × 10^9^/L (range: 2.66–9.51 × 10^9^/L). The median LYM count upon admission was 1.09 × 10^9^/L (range: 0.38–3.72 × 10^9^/L), with 57.1% of the patients having a LYM count below normal. The LYM count significantly increased during hospitalization and the median LYM count at discharge was 1.47 × 10^9^/L (range: 1.10–4.11 × 10^9^/L). The LDH level was higher than normal in 52.2% of the patients upon admission, with a median of 225.22 U/L (range: 153.48–576.53U/L). LDH had returned to normal levels upon discharge with a median of 183.67U/L (range: 131.20–233.89 U/L). The CRP level was higher than normal in 75.7% of the patients upon admission, with a median of 20.43U/ML (range 0.37–121.22 U/ML). CRP had returned to normal levels upon discharge, with a median of 2.07U/ML (range: 0.25–8.12U/ML). The ESR was higher than normal in 60.0% of the patients upon admission, with a median of 30.00 mm/h (range 3.01–220.00 mm/h). The ESR had decreased upon discharge, with a median of 19.00 mm/h (range 0.18–20.90 mm/h) at discharge. A significant difference was observed between the temperature and the laboratory test results upon admission and upon discharge (*P* < 0.05) (Table 2).

**Table 2:** Fever and laboratory values.

	Admission	Discharge	*P* value
Body temperature (℃)	37.8 (36.0–39.6)	36.5 (36.0–37.2)	0.000
WBC (10^9^/L)	4.52 (0.83–14.69)	5.69 (2.66–9.51)	0.000
LYM (10^9^/L)	1.09 (0.38–3.72)	1.47 (1.10–4.11)	0.000
LDH (U/L)	225.22 (153.48–576.53)	183.67 (131.20–233.89)	0.000
CRP (U/ML)	20.43 (0.37–121.22)	2.07 (0.25–8.12)	0.000
ESR (mm/h)	30.00 (3.01–220.00)	19.00 (0.18–20.90)	0.004

The body temperature, WBC, LYM, LDH、CRP、ESR at discharge, excluding the 2 deaths. *p* < 0.05 was considered statistically significant.

### Chest CT image analysis results

**Figure 1:**
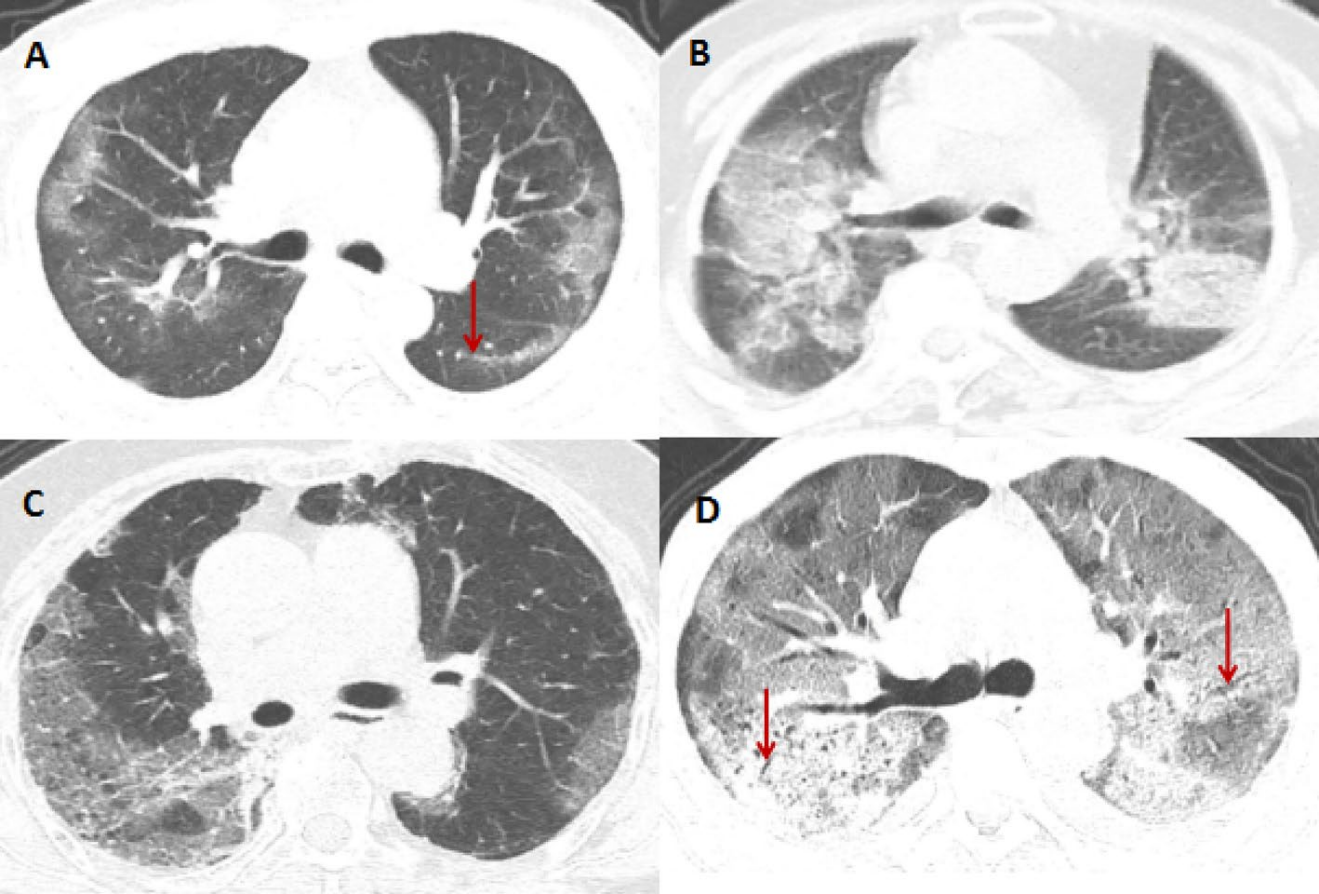
Main image manifestations and accompanying signs. (**A**) 34-year-old man; the main sign is bilateral subpleural ground glass opacities with left lower lung dorsal linear opacities (arrow); (**B**) 68-year-old man; the main signs are bilateral ground glass opacities and large consolidation; (**C**) 74-year-old woman; the main signs are bilateral ground glass opacities and reticular patterns, “crazy-paving” pattern, thickened small vessel shadows can be seen in the right lung lesions; (**D**) 59-year-old man; extensive lung glass shadow and partial consolidation shadow; lungs are white; air bronchial signs (arrows) can be seen in the background of bilateral exudation, and the dorsal pleura is slightly thicker. Imaging were acquired on GE AW VolumeShare software (Version number: 4.6; http://www.gehealthcare.com/products/advanced-visualization/platforms/aw-server).

**Figure 2:**
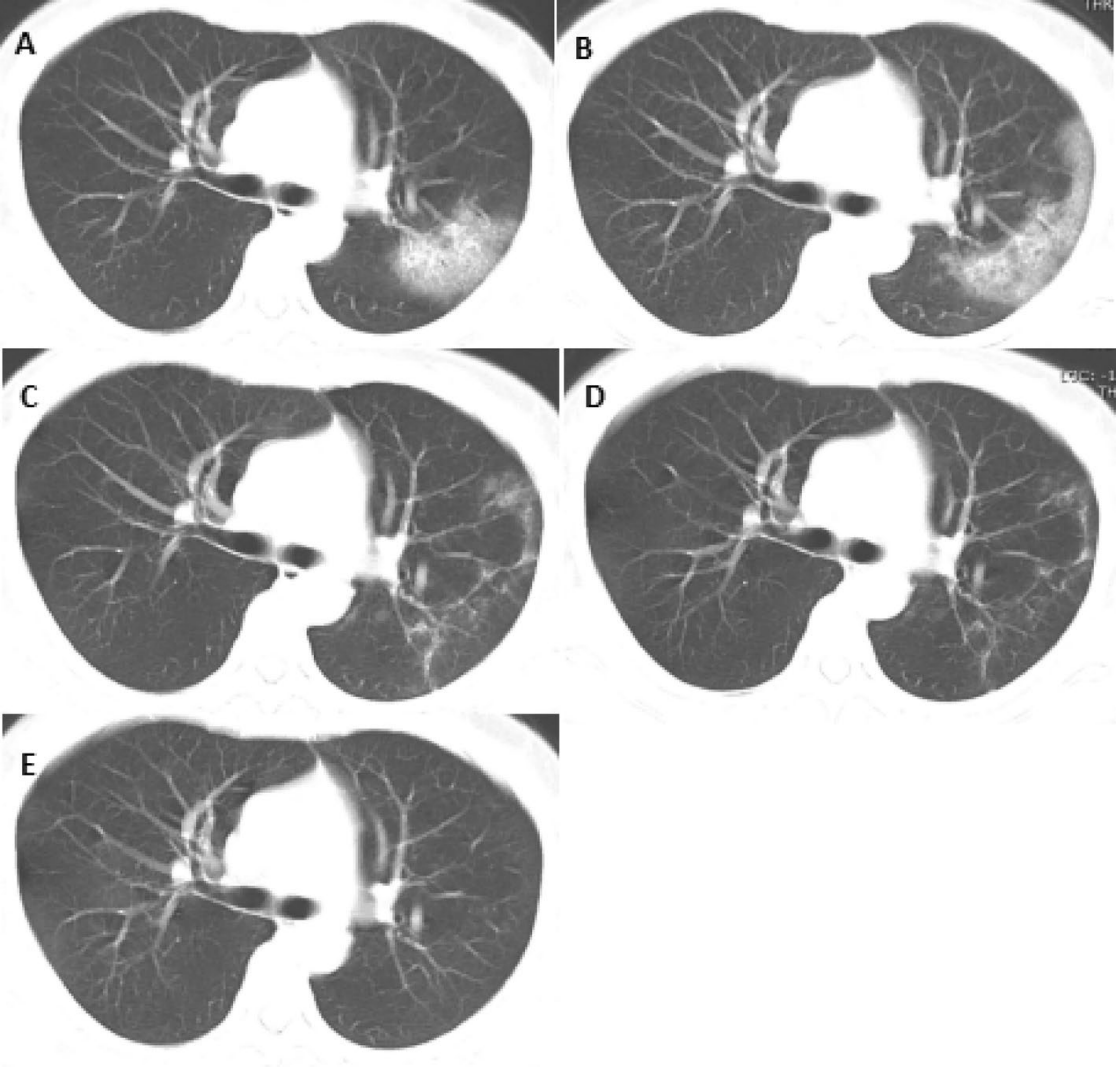
Case of complete absorption of lung lesions. A 44-year-old female patient was hospitalized for 23 days and her lung lesions were gradually absorbed to disappearance. (**A**) The first CT on admission (2020–1–27): flaky ground glass on the outside of the right lung; (**B**) The second CT (2020–1–31): progression of the right lung lesion, expanded scope; (**C**) Third CT (2020–2–05): obvious absorption of right lung lesions, visible small plate-shaped ground glass opacities and stripe shadows; (**D**) Fourth CT (2020–2–12): the right lung lesions are further absorbed and reduced; (**E**) Fifth CT (2020–2–19): Complete absorption of the right lung lesion. Imaging were acquired on GE AW VolumeShare software (Version number: 4.6; http://www.gehealthcare.com/products/advanced-visualization/platforms/aw-server).

The patients had a median of 5 chest CT scans during the hospital stay. The most serious pulmonary inflammation on each patient’s chest CT was selected for analysis and scoring. Among the 118 patients, the chest CT from 1 patient showed no pathological changes in the lungs, while the chest CT from the other 117 patients showed inflammatory lesions in the lungs, with a median of 4 (range: 0–5) lobes and a median of 9 (range: 0–20) lung segments involved. The inflammatory pathological changes were most likely to be found in the left and right lower lobes of the lungs (89.0% and 83.9%), and 84.7% of them had inflammatory pathological changes in both lungs. The main manifestations found on chest CT were as follows: ground glass opacities (Fig. 1A) (31.4%), ground glass opacities and consolidation (Fig. 1B) (20.3%), ground glass opacities and reticular patterns (Fig. 1C) (32.2%), mixed type (13.6%), and white lungs (Fig. 1D) (1.7%). Common secondary signs present on chest CT included the following: linear opacities (55.9%) (Fig. 1A), air bronchograms (46.6%) (Fig. 1D), thickened small blood vessel shadows (36.4%) (Fig. 1C), and pleural hypertrophy (13.6%) (Fig. 1D). Rare secondary signs identified on chest CT were as follows: nodular shadows, mediastinal lymph node enlargement, and pleural effusion. Upon discharge, chest CT showed that the inflammatory lesions had been completely absorbed in 19 cases (16.1%) (Fig. 2), but the inflammatory lesions in 329 lobes or 600 segments of the lungs in the remaining 99 cases had still not been completely absorbed and dissipated. Inflammatory lesions remained in a median of 3 lobes (range: 0–5) and a median of 5 lung segments (range: 0–20). The residual lesions mainly presented as ground glass opacities (61.0%), and the main secondary signs were strip linear opacities (59.3%)(Table 3).

**Table 3:** Manifestations on chest CT scans of 118 patients at admission and discharge.

	The most severe CT manifestations	Discharge CT manifestations	*P* value
**Pulmonary involvement**			
Number of lung lobes involved	438	329	0.000
No involvement	1 (0.8%)	19 (16.1%)	0.000
Double lung involvement	100 (84.7%)	84 (71.2%)	0.012
Right upper lobe	79 (66.9%)	56 (47.5%)	0.002
Right middle lobe	72 (61.0%)	42 (35.6%)	0.000
Right lower lobe	99 (83.9%)	81 (68.6%)	0.006
Left upper lobe	83 (70.3%)	64 (54.2%)	0.011
Left lower lobe	105 (89.0%)	86 (72.9%)	0.002
Median of the involved lung lobes	4 (0–5)	3 (0–5)	0.000
Number of lung segment involvement	1066	600	0.000
Median of the involved lung segment	9 (0–20)	5 (0–20)	0.000
**Imaging signs**			
**Main signs**			
No performance	1 (0.8%)	20 (16.9%)	0.000
Ground glass opacities	37 (31.4%)	72 (61.0%)	0.000
Ground glass opacities and consolidation	24 (20.3%)	12 (10.2%)	0.030
Ground glass opacities and reticular patterns	38 (32.2%)	10 (8.5%)	0.000
Mixed type	16 (13.6%)	2 (1.7%)	0.001
White lung	2 (1.7%)^**※**^	2 (1.7%)^**※**^	1.000
**Secondary signs**			
None	17 (14.4%)	44 (37.3%)	0.000
Linear opacities	66 (55.9%)	70 (59.3%)	0.598
Bronchograms	55 (46.6%)	11 (9.3%)	0.000
Thickness of small blood vessels	43 (36.4%)	6 (5.1%)	0.000
Pleural hypertrophy	16 (13.6%)	12 (10.2%)	0.421
Nodular shadow	5 (4.2%)	4 (3.4%)	0.734
Mediastinal lymph node enlargement	2 (1.7%)	0 (0.0%)	0.156
Pleural effusion	1 (0.8%)	1 (0.8%)	1.000
**PII**	30.0% (0–97.5%)	12.5% (0–53.0%)^**Δ**^	0.000

*p* < 0.05 was considered statistically significant. ※Two patients showed severe white lung disease. After treatment, there was no improvement, and the symptoms remained. The two patients eventually died. **Δ** Statistics exclude two deaths.

A total of 583 CT scans corresponded to 583 PIIs, the largest PII of each patient was selected for scoring, with the median maximal PII at 30.0% (range: 0–97.5%). The median PII for patients upon discharge was 12.5% (range: 0–53.0%). A significant statistical difference was observed between the maximum PII and the PII upon discharge (*P* = 0.000) (Table 3).

### The reliability and reproducibility of CT findings

**Figure 3:**
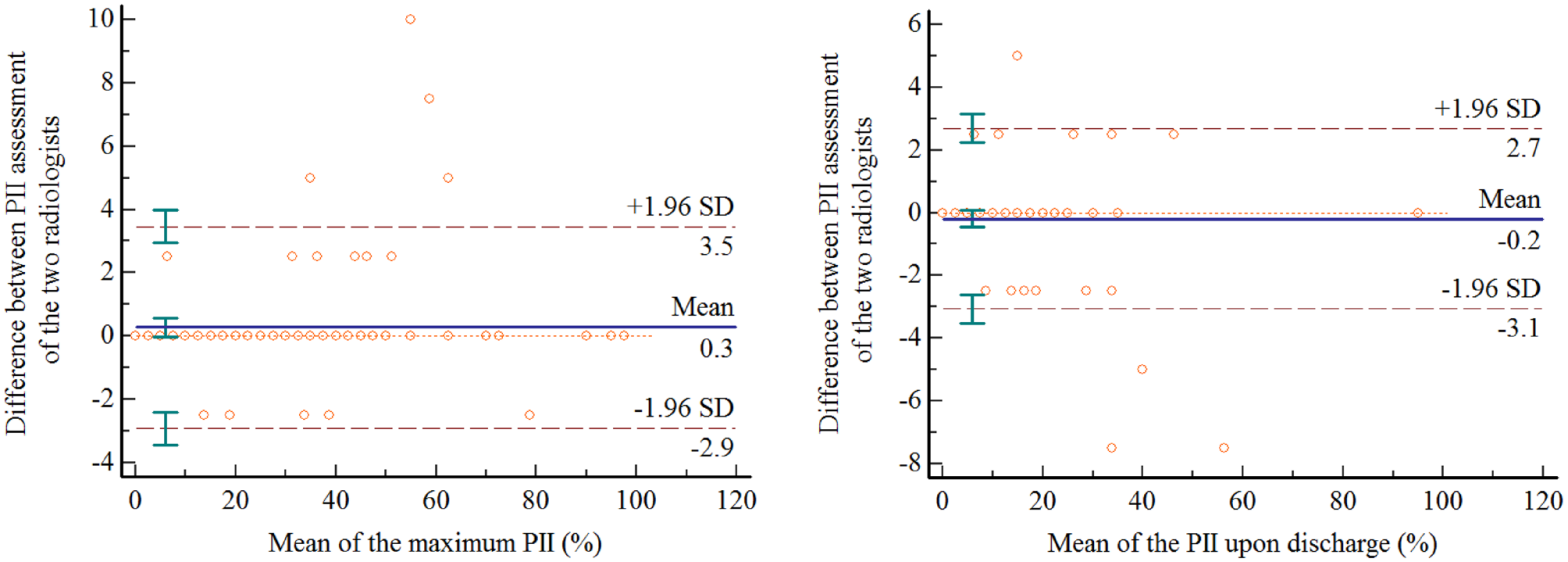
The reliability of quantitative methods for PII between Radiologist 1 and Radiologist 2. The left hand panel depicts the Bland–Altman plot of the two radiologists assessed the maximum PII. The right hand panel depicts the Bland–Altman plot of the two radiologists assessed the PII upon discharge. The upper and lower dashed lines represented the 95% confidence intervals.

As shown in Fig. 3, the Bland–Altman plot displayed excellent agreement in maximum PII and PII upon discharge from hospital of the patients between radiologist 1 and 2. Therefore, these plots demonstrated that the reliability of quantitative methods was high.

### Relationship between PII and clinical classification

**Figure 4:**
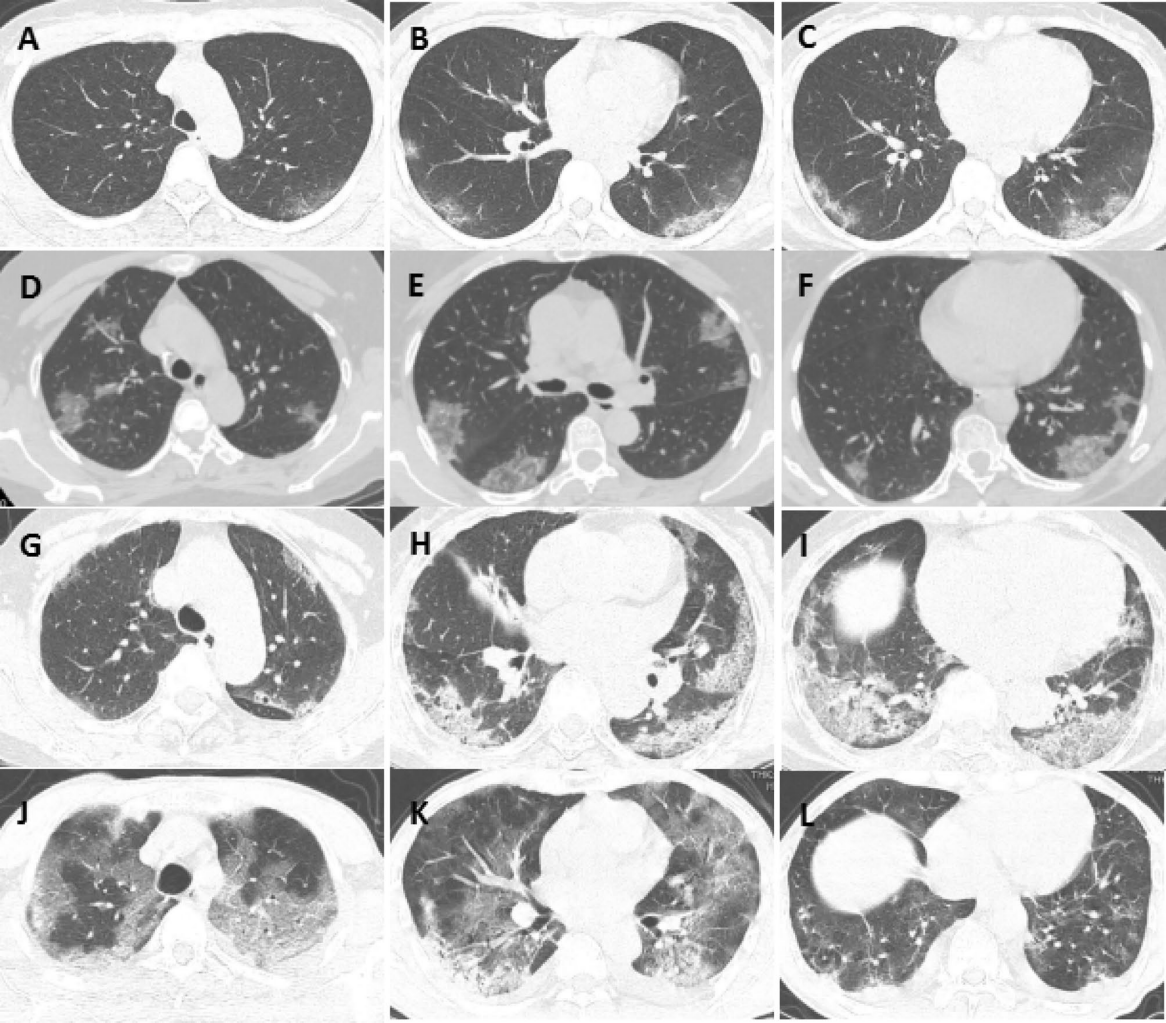
Grade and clinical classification of inflammatory factors. (**A**–**C**) 41-year-old female, ground glass opacities in both lungs, involving 5 lung segments, PII was 12.5%, inflammation grade I, clinically classified as moderate type; (**D**–**F**) 56-year-old female, scattered lung glass shadows, involving 13 lung segments, PII was 37.5%, degree of inflammation was grade II, clinically classified as moderate type; (**G**–**I**) 41-year-old female, double lung ground glass opacities with consolidation and thickened lobular septa, the image showed mixed type, involving 16 lung segments, PII is 62.5%, the degree of inflammation is grade III, clinically classified as severe type; (**J**–**L**) 57 years old, bilateral ground glass opacities combined with consolidation and stripe shadow, bilateral pleural effusion, involving 16 lung segments, PII is 77.5%, the degree of inflammation is grade IV, clinically classified as severe type. Imaging were acquired on GE AW VolumeShare software (Version number: 4.6; http://www.gehealthcare.com/products/advanced-visualization/platforms/aw-server).

The patients in this study were classified into 5 levels according to the range of PII: Grade 0 (PII = 0%); Grade I (PII = 1–25%); Grade II (PII = 26–50%); Grade III (PII = 26–50%); and Grade IV (PII = 76–100%) (Fig. 4).

Among the 118 patients, the 1 case classified as mild corresponded to grade 0; of the 111 cases classified as moderate, 50 cases (45.0%) corresponded to grade I, 54 cases (48.7%) corresponded to grade II, and 7 cases (6.3%) corresponded to grade III; of the 4 classified as severe, 2 cases corresponded to grade III, and 2 cases corresponded to grade IV; the 2 cases classified as critical corresponded to grade IV (Table 4). The PII was significantly negatively correlated with the lymphocyte count (*P* = 0.000) and significantly positively correlated with body temperature (*P* = 0.000, R = 0.472), LDH (*P* = 0.000, R = 0.530), CPR (*P* = 0.000, R = 0.631), and ESR (*P* = 0.000, R = 0.665). We did not find a significant relationship between the PII and the white blood cell count (*P* = 0.271).

**Table 4:** Clinical classification corresponding to the grade of maximum PII.

	Grade 0 (PII = 0%)	Grade I (PII = 1–25%)	Grade II (PII = 26–50%)	Grade III (PII = 51–75%)	Grade IV (PII = 76–100%)
Mild type	1				
Moderate type		50	54	7	
Severe type				2	2
Critical type					2

## Discussion

SARS-CoV-2 is a new coronavirus belonging to the genus β. It is the seventh member of the coronaviridae family known to infect humans^10^. It is an enveloped virus with round or oval particles, often polymorphic. The virus’s size measures between 60 and 140 nm in diameter. SARS-CoV-2 is mainly transmitted through respiratory tract and contact, and the virus invades the interior of the cell through receptor binding with the angiotensin-converting enzyme II (ACE II) of mucosal cells^11^. So far, the mortality rate of COVID-19 pneumonia is reported to be lower than severe acute respiratory syndrome (SARS) or Middle East respiratory syndrome (MERS) coronavirus diseases^12–14^; however, SARSCoV2 is highly contagious and can pose a major threat to health^15^. It is very important to control the spread of the disease, improve the recovery rate and reduce the death rate for patients with COVID-19 pneumonia. Patients should be treated in isolation as early as possible and treatment plans should be adjusted based on ongoing clinical findings. Chest CT can be used not only for the screening of suspected COVID-19 pneumonia patients, but also to confirm the diagnosis as soon as possible. It also plays an important role in the evaluation of the disease and the judgment of discharge using imaging standards.

At present, there are relatively few reports on the relationship between COVID-19 pneumonia chest CT manifestations and disease course. This study shows that there is no difference in patients' gender and age, which is consistent with previous studies^16,17^. The age range of patients in this study was from 1 to 74 years, indicating that anyone could be susceptible to infection. Previous studies have suggested that most patients have good prognosis, finding that only a low percentage of the patents were in critical condition, but also noted that the elderly and those with underlying disease comorbidities had a poor prognosis^5,18,19^. In this study, 116 cases recovered and only 2 patients died, with a mortality rate of 1.7%. Our results were similar to the previous studies in the prognosis of COVID-19 pneumonia. The two patients who died were 68 and 39 years old. The 39-year-old patient had diabetes and chronic renal failure, but the 68-year-old patient did not have other diseases. “White lungs” were observed in two of them on the chest CT.

COVID-19 pneumonia is clinically characterized by fever, dry cough, and fatigue. Lymphocyte counts were generally lower than normal and the CRP levels and ESR were higher in most cases^20–22^. The present study showed that 76.3% of the patients had fever with a median body temperature at admission of 37.8 ℃, ranging from 36.0 to 39.6℃. After treatment, the temperature returned to normal, and the median temperature at discharge was 36.5℃. Our results also showed that the average lymphocyte count was below the normal range, and the average LDH level, CPR level, and ESR were at higher than normal values when the patients were admitted to the hospital, which all returned to normal upon patient discharge from the hospital. The previous studies have shown that the decrease in lymphocyte counts indicates that the occurrence and development of COVID-19 may be similar to the pneumonia caused by SARS and MERS coronavirus. There may be a process of cellular immunity impairment, which is a common feature of COVID-19 patients and may be related to the severity of the disease and the key factors related to mortality^23,24^. The increase of LDH indicates that that SARS-CoV-2 virus may damage cardiomyocytes and hepatocytes directly or indirectly through cytokines. In addition, CRP levels and ESRs reflect the size or activity of inflammatory tissue, and have a good correlation with disease activity during acute inflammation and infection. Therefore, the lymphocyte count, LDH level, CPR level, ESR and body temperature can be used to help make the diagnosis of COVID-19 pneumonia and to assess the effects of therapy.

The Radiological Branch of the Chinese Medical Association recommended a time window of 3–7 days for CT reexamination of patients with COVID-19 pneumonia. In this study, the median hospital stay for the patients was 22 days (range: 9–41 days), the median number of CT examinations during hospitalization was 5 (range: 3–6) times, and the median time between CT examinations is 4.5 days (range: 3–8 days). The imaging manifestations of COVID-19 pneumonia are similar to SARS and MERS, and are mainly characterized by ground glass opacities and lung consolidation^25–29^. In the early stage of the onset of the disease, chest CTs often show multiple lesions in both lungs, and most of the lesions are located in the periphery of the lungs. As the disease progresses, the range of ground glass opacities increases, the lesions become lobular or diffuse in the lungs, and the bronchial wall and the bronchovascular bundle thicken. The lesions presented in bilateral asymmetrical wedge-shaped or fan-shaped distributions, and were more common in the subpleural space in the lung base and dorsal area, with some distributed along the bronchovascular bundles. The ground glass opacities’ densities increased with the increase of exudate in the alveoli. Consolidation can be seen as a density shadow of soft tissue or a dense stripe shadow, and distributed either segmentally or lobularly. The CT features of severe and critical patients mostly presented as diffuse lesions of the lungs, and some of them presented as "white lungs". The lesions are mainly consolidated, combined with ground glass density shadows, air bronchogram signs, and multiple hairline shadows.

During the recovery period, the pulmonary function of the patients improved. The chest CT showed that lesion size was reduced, density was decreased, the exudate was absorbed, and the consolidation lesion was gradually dissipated. The lesions evolved into a ground glass-like shadow, which either disappeared completely or became a stripe shadow remnant.

Among the 118 patients in this study, one patient did not have any symptoms with normal laboratory test results, and no changes on chest CT. The remaining 117 patients had symptoms, and the chest CTs showed that the lower lobes in both lungs were most likely involved, and that often both lungs were involved at the same time. The main manifestations on chest CT were consistent with other studies^16–20^. After a median of 22 days of hospital stay, 116 patients reported that symptoms disappeared, body temperature and laboratory results returned to normal, and the two-time nucleic acid tests were negative. However, chest CTs showed only 19 cases (16.1%) of complete absorption at discharge. Although the lesions were significantly absorbed in the remaining 97 cases, ground glass opacities and/or stripe shadows were seen on the chest CT. Therefore, we surmised that the residual ground glass opacities could be completely absorbed over time, but further follow-up after discharge is required to explore if the remaining linear opacities could be completely absorbed.

In contrast to the volumetric algorithm or the semi-quantitative method of lung lobe assessment in the previous studies, this study evaluated PII and scored each involved segment of the lungs, which makes it more accurate in quantifying the extent of lung inflammation. The results of this study showed that the lung PII is negatively correlated with the lymphocyte count and positively correlated with body temperature, LDH level, CPR level, and ESR, demonstrating that chest CT can be used to accurately determine the degree of inflammation of COVID-19 pneumonia. We also found that the grade of inflammatory factors has a strong correlation with the clinical classification. Therefore, PII can be used to reflect the severity of the disease and assist in clinical classification and treatment plan adjustment.

This study is limited because this is a retrospective study. Firstly, the onset of this disease was not recorded clearly in every case, making it difficult to comprehensively analyze the entire process of this disease. Secondly, the laboratory tests and CT scans were not performed on the same day, so it may affect the correlation between PII and chest CT and laboratory results. Finally, this study lacked the CT and laboratory results after the patients were discharged from the hospital. Since nucleic acid detection tests may be false negatives, it is necessary to perform the chest CT to follow up on the changes of the inflammatory lesions after the patients were discharged from the hospital.

## Conclusion

In summary, the main manifestations on chest CT of patients with COVID-19 are ground glass opacities, ground glass opacities and consolidation, ground glass opacities and reticular patterns, mixed type, or white lungs; common secondary signs included linear opacities, air bronchograms, thickened small blood vessel shadows and pleural hypertrophy. Body temperature, lymphocyte count, LDH level, CPR level, and ESR are useful in the diagnosis of COVID-19 pneumonia and in judgment of therapeutic efficacy. Body temperature, lymphocyte count, LDH level, CPR level, and ESR returned to normal earlier than the inflammatory changes on the chest CT. The quantitative PII can be used to reflect the severity and treatment outcome of COVID-19 pneumonia, and provide help for clinical classification and adjustment of the treatment plan.
